# Searching HPV genome for methylation sites involved in molecular progression to cervical precancer

**DOI:** 10.7150/jca.30081

**Published:** 2019-08-07

**Authors:** Christine Kottaridi, Danai Leventakou, Abraham Pouliakis, Vasileios Pergialiotis, George Chrelias, Eugenia Patsouri, Andriani Zacharatou, Eleni Panopoulou, Vasileia Damaskou, Vasileios Sioulas, Charalambos Chrelias, Sofia Kalantaridou, Ioannis G. Panayiotides

**Affiliations:** 1 2 nd Department of Pathology, University General Hospital “ATTIKON”, School of Medicine, National and Kapodistrian University of Athens, Athens 12464, Greece; 2 3 rd Department of Gynaecology and Obstetrics, University General Hospital “ATTIKON”, School of Medicine, National and Kapodistrian University of Athens, Athens 12464, Greece

**Keywords:** HPV16, methylation, *UTR*, * E6*, *E7*

## Abstract

**Background:** Human Papilloma Virus has been considered as the main cause for cervical cancer. In this study we investigated epigenetic changes and especially methylation of specific sites of HPV genome. The main goal was to correlate methylation status with histological grade as well as to determine its accuracy in predicting the disease severity by establishing optimum methylation cutoffs.

**Methods**: In total, sections from 145 cases genotyped as HPV16 were obtained from formalin- fixed, paraffin-embedded tissue of cervical biopsies, conization or hysterectomy specimens. Highly accurate pyrosequencing of bisulfite converted DNA, was used to quantify the methylation percentages of *UTR* promoter, enhancer and 5' UTR, *E6* CpGs 494, 502, 506 and *E7* CpGs 765, 780, 790. The samples were separated in different groupings based on the histological outcome. Statistical analysis was performed by SAS 9.4 for Windows and methylation cutoffs were identified by MATLAB programming language.

**Results:** The most important methylation sites were at the enhancer and especially *UTR* 7535 and 7553 sites. Specifically for CIN3+ (i.e. HSIL or SCC) discrimination, a balanced sensitivity vs. specificity (68.1%, 66.2% respectively) with positive predictive value (PPV) and negative predictive value (NPV) (66.2%, 68.2% respectively) was achieved for *UTR* 7535 methylation of 6.1% cutoff with overall accuracy 67.1%, while for *UTR* 7553 a sensitivity 60.9%, specificity 69.0%, PPV=65.6%, NPV=64.5% and overall accuracy=65.0% at threshold 10.1% was observed.

**Conclusion:** Viral HPV16 genome was found methylated in NF-1 binding sites of *UTR* in cases with high grade disease. Methylation percentages of *E6* and *E7* CpG sites were elevated at the cancer group.

## Introduction

Cervical cancer is the fourth most common cancer in women, and the seventh overall, with an estimated 528,000 new cases in 2012. A large majority (around 85%) of the global burden occurs in the less developed regions, where it accounts for almost 12% of all female cancers. Since 1999, Human Papilloma Virus (HPV) has been considered as the main cause for cervical cancer 1. Over 100 different types of the virus have been isolated from human samples, out of which 20 are considered oncogenic and have been epidemiologically linked to this cancer 2.

A persistent infection by HPV aided by other parameters results in HPV integration and progression to high grade lesions. Most HPV infections are cleared by the host's immune system before they progress to lesions. Prophylactic HPV vaccines and traditional Pap-smear screening are undoubtedly capable of decreasing the incidence and mortality of cervical cancer. However, a large number of females succumb to the disease each year due to late diagnosis and resistance to conventional treatments. Nowadays, the more extensive use of modern molecular biological methods, have added to cervical cancer screening approaches 3.

In order to understand the biology of this tumor, it is of utmost importance to analyze its molecular dynamics aiding to improve the clinical outcome. Epigenetics is a well-established phenomenon that plays a major role in virus-associated neoplasms 4, 5 and one of the most widely studied epigenetic changes is DNA methylation. During cervical carcinogenesis, substantial changes in methylation are observed in both the host cell and the viral genome. Methylation of the viral DNA has been recently proposed as a novel biomarker with encouraging results 6. The quantification of the percentage of cytosines with a covalently added methyl-group at individual CpG (Cytosine—phosphate—Guanine) dinucleotides reflects the degree of epigenetic changes of the viral genome. Many studies mainly focused on *L1* viral gene, have already shown that the methylation percentage of HPV 16 specific CpG sites along this viral gene, is increasing gradually and it is highest in women with high-grade cervical neoplasia 7-10.

This study aims to assess whether quantitative measurement of methylation of CpGs along the HPV 16 *UTR*, *E6* and *E7* genes, could predict the presence of high-grade disease at histology in women testing positive for the HPV 16 genotype. More specifically, we aim to correlate specific sites with the histological grade and to determine their accuracy in predicting the disease severity by establishing optimum methylation cutoffs.

## Material and Methods

In total, sections from 145 non-pregnant women, 21-62 years of age genotyped as HPV16 were obtained from formalin-fixed, paraffin-embedded tissue of cervical biopsies, conization or hysterectomy specimens of females that visited the gynecology clinic of Attikon University General Hospital, Athens, Greece, between May 2014 and May 2016. Women were included irrespective of their ethnicity, smoking habits, phase in their cycle, menopausal status and contraceptive practices. Women who were HIV or hepatitis B/C positive, with autoimmune disorders, or had a previous history of cervical treatment were excluded. The histological diagnoses included the following groups: normal, CIN1, CIN2, CIN3, squamous cell carcinoma (SCC), and adenocarcinomas.

DNA was extracted from formalin-fixed, paraffin-embedded tissue sections, using QIAamp DNA FFPE Tissue Kit (Qiagen, Heidelberg Germany). All steps in the purification procedure were done using the automated QIAcube technology (Qiagen, Heidelberg Germany). DNA typing was performed with the HPV Genotypes 14 Real-TM Quant (Sacace Biotechnologies, Como Italy) for the quantitative detection and genotyping of Human Papillomavirus types (16, 18, 31, 33, 35, 39, 45, 51, 52, 56, 58, 59, 66 and 68). The extracted DNA concentrations were measured with QIAexpert technology (Qiagen, Heidelberg Germany).

DNAs were then bisulfite converted using the EpiTect Bisulfite Kit (Qiagen, Heidelberg Germany), according to the manufacturer's instructions and stored at -80°C. Biotin-labeled primer sets, sequencing primers and polymerase chain reaction conditions were used as previously described 11 to amplify HPV16 *UTR* region, while biotin-labeled primer sets and sequencing primers for *E6* and *E7* genomic regions were designed in the present study with the aid of PyroMark Assay Design 2.0 (table 1). PCR conditions for *E6* amplification were preheating at 95 °C for 5 min, 40 cycles at 95 °C for 30 s, 53 °C for 30 s, 72 °C for 30 s, and a final extension at 72 °C for 10 min, while for *E7* amplification were 95 °C for 5 min, 40 cycles at 95 °C for 30 s, 58 °C for 30 s, 72 °C for 30 s, and a final extension at 72 °C for 10 min. The methylation quantification was performed by Pyrosequencing technology (PyroMark Q24, Qiagen, Heidelberg, Germany), which provides a site-specific quantification of methylation at individual CpG sites. Pyrosequencing protocols were at first applied to bisulfite converted SiHa cells. This cervical cell line contains a single genome of HPV-16 integrated into chromosomal DNA which is completely unmethylated at *LCR*, *E6* and *E7* genomic regions 12 a finding that was confirmed by our protocols. In order to establish a limit of blank for each specific methylation site, we performed series of ten measurements using SiHa DNA. The highest value that was reported in the series of measurement was 3.4%. DNA from SiHa cells was run in every experiment as an unmethylated control. As of lack of an artificially methylated control for the studied CpG dinoucleotides spanning along HPV16 genome, we randomly chose a highly methylated to all sites clinical sample and performed ten independent experiments to check the reproducibility of our protocols to detect methylation. The measurements had at most 3.2% standard deviation around the mean value. The *UTR* methylation analysis included CpGs sites which are located within the p97 HPV 16 promoter (31, 37, 43, 52, 58, 7862), the enhancer (7535, 7553) and the 5'*UTR* (7270, 7461, 7455, 7428). The *E6* methylation analysis included CpGs 494, 502 and 506, while the *E7* methylation analysis included CpGs 765, 780 and 790 (reference sequence NC_001526).

### Statistical analysis

The statistical analysis was performed by programming in SAS 9.4 for Windows (SAS Institute Inc. NC, USA) 13, 14. Microsoft Excel for Windows was used for data storage and preprocessing. We applied the student's t-test to examine if the various methylation percentages were statistically different between various histological groupings. Finally, the algorithms for the determination of the optimum threshold values were developed in-house within the MATLAB software environment and programming language (The MathWorks, Inc. Natick, Massachusetts, U.S.A.).

We calculated different accuracy parameters for the ability of the mean methylation to detect the presence of disease for the previous histological cut- offs. These included the sensitivity (Sens), specificity (Spec), positive (PPV) and negative predictive value (NPV), the false positive (FPR) and false negative (FNR) rate, the overall accuracy (OA) and the positive (PLR) and negative likelihood ratio (NLR). These parameters were calculated from methylation percentage 0% and increased up to 100% using an increment step of 0.1%, as described in our previous studies 7, 15-17. Subsequently graphs depicting sensitivity, specificity and overall accuracy for all methylation positions were produced and the above measures were reported for two positions, i.e. the methylation position that maximizes OA and the position that produces a more balanced result, i.e. whereas the difference between sensitivity and specificity is minimal.

In order to identify any possible correlations of age or disease severity with the methylation levels and the mean methylation per region it was used the Spearman correlation coefficient (R_s_). Specifically concerning disease severity the histological status was numerically coded as 1 to 5 for the histological categories of: normal, CIN-1, CIN2, CIN-3 and carcinomas (SCC and adenocarcinomas) respectively.

## Results

### Study group profile

Age at diagnosis ranged from 21 to 62 years, with a mean of 35.52 years (SD: ±8.35) and a median of 34 years. Out of the 145 studied cases 69 (47.59%) had single HPV16 infection and from the remaining cases with multiple infections, 50 cases (34.48%) had only high risk infections and 26 (17.93%) cases had high risk and low risk infections. Multiple infections were more frequent in negative and CIN1 cases, specifically 71.43% (n=5) from negative cases and 60.87% (n=14) from CIN1 had multiple infections; for CIN2 and CIN3 the percentages of multiple infections were 53.33% (n=24) and 52.46% (n=32) respectively (Table 2). Finally out of the 6 SCC cases, 5 (83.33%) had single HPV16 infection, and similarly all three cases of adenocarcinomas.

### Methylation analysis

**Figure 1:**
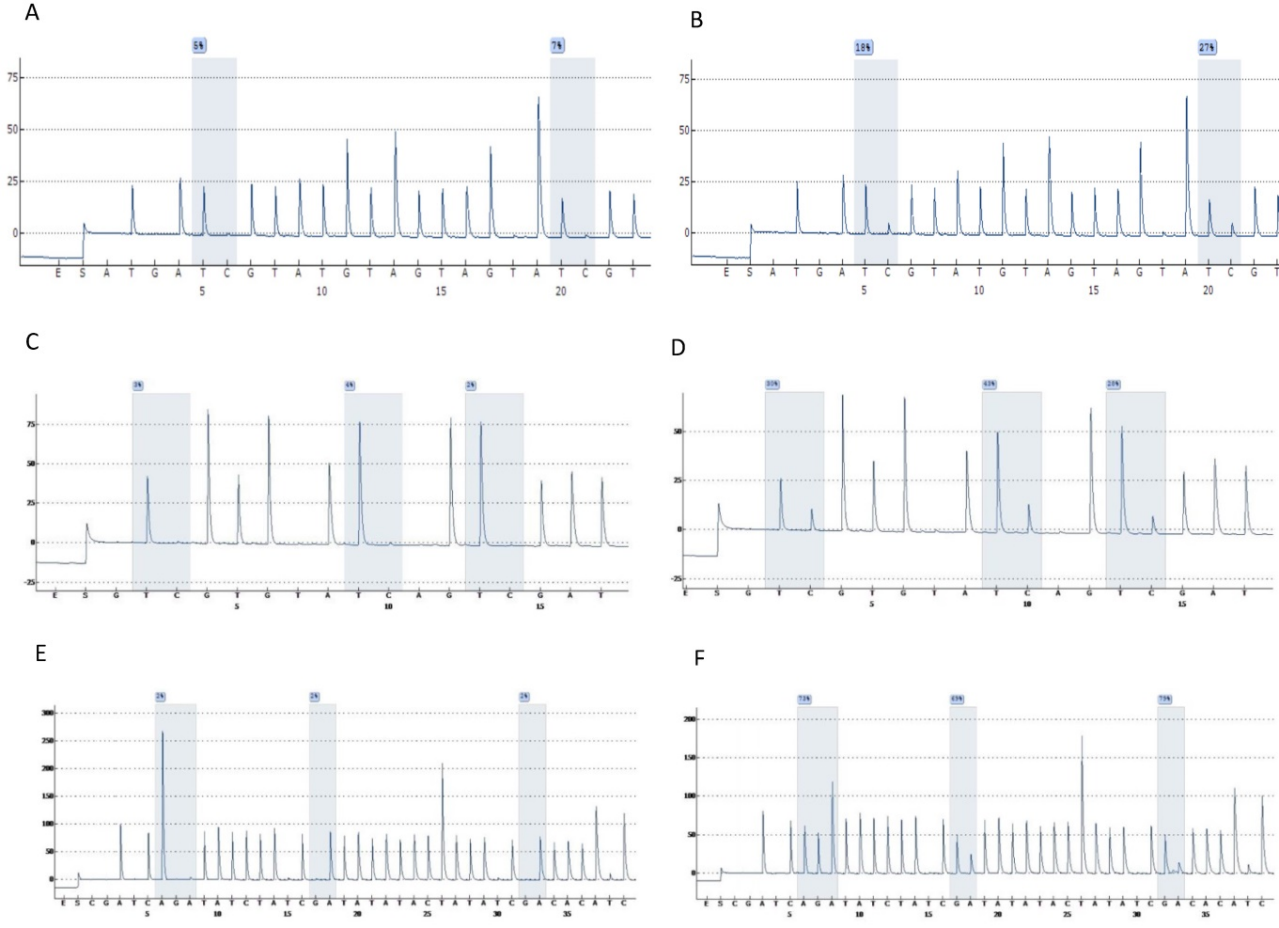
Pyrograms depicting unmethylated (left column) and methylated samples (right column). Grey blocs are presenting the under investigation CG sites and numbers at blue squares the methylation percentages. A & B, *UTR* genomic region sites 7535, 7553. C & D, *E6* genomic region sites 494, 502, 506. E & F, *E7* genomic region sites 765, 780, 790 (a reverse primer was used for pyrosequencing of this region).

Highly accurate pyrosequencing technology which performed after bisulfite treatment of DNA was used to calculate methylation percentage of specific sites harbored at 5'*UTR*, enhancer and promoter of the viral genome (nt 7270, 7461, 7455, 7428, 31, 37, 43, 52, 58, 7862) as well as the *E6* (494, 502, 506) and *E7* (765, 780, 790) gene. At figure 1 are presented pyrograms depicting samples either methylated or unmethylated at the genomic regions that were studied.

The mean methylation of 5' *UTR* (7270, 7461, 7455, 7428) CpG sites between the different histological groups was (Table 3) 5.6±3.4, 5.3±4.0, 5.8±2.5, 6.6±3.3, 13.3±13.1 and 14.5±8.3 (for Normal, CIN-1, CIN-2, CIN-3, SCC and adenocarcinomas respectively). The two CpG sites (7535, 7553) mapped at the enhancer of the genome, had a mean methylation that ranged from 6.3±3.2 to 39.2±19.2 when the studied groups were analyzed (Table 3). Respectively, the p97 promoter CpG sites (7862, 31, 37, 43, 52, 58) depicted methylation levels, varying from 1.4±0.5 to 9.6±13.2 (Table 3).

The methylation of *E6* CpG sites ranged from 8.0±6.9 to 40.6±27.8 among the histological groups (table 3) and the methylation of *E7* CpG sites ranged 3.6±1.8 to 29.6±27.2 among the various histological groups (table 3).

### Methylation and age

Concerning the relation of the 5' *UTR* region sites analyzed in this study the analysis via Spearman correlation indicated that no specific site neither the mean methylation for all 5' *UTR* sites was related to the women age. Similarly, for the enhancer region it was found a rather low correlation (R_s_=0.23, p=0.0053) of age with site 7535, while the correlation for site 7553 was R_s_=0.15 (p=0.0802), and for the mean methylation of the enhancer region was R_s_=0.20, p=0.0248. The correlation of the promoter sites with age showed a weak correlation with methylation levels at site 7862 (R_s_=0.18504, p=0.0280) and there was no correlation of the remaining sites or of the mean methylation of the promoter region. Furthermore, no significant relation of age with the methylation of sites from *E6* or *E7* region, neither the mean methylation of the *E6* or *E7* region were identified (p>0.05 for all cases).

### Methylation and disease severity

The statistical tests correlating disease severity (as formed in a numeric manner) with methylation percentages indicated generally weak correlations. Specifically, for the 5'*UTR* sites the correlation for sites 7270, 7461, 7428 and the mean methylation of all 5' *UTR* sites was R_s_ 0.22, 0.36, 0.24 and 0.29 respectively, p<0.05, while for sites 7535, 7553 and for the mean enhancer methylation a correlation of 0.33, 0.29 and 0.35 respectively, p<0.001 was found. A negative correlation for site 43 of the promoter (R_s_ -0.23, p=0.0052) was indicated. As far as *E6* is concerned, a weak correlation existed for sites 494, 502 and the mean *E6* methylation (R_s_ 0.18, 0.20 and 0.17 respectively, p<0.05) and finally for site 790 of the *E7* region the correlation was 0.19, p=0.0265.

### Calculation of thresholds discriminating between histological groups and performance characteristics

Accuracy parameters were determined for different histological cut-offs. We searched for a methylation threshold optimizing overall accuracy as well as for a threshold that produces a balanced sensitivity vs. specificity (table 4). The most important identified methylation sites were on the enhancer region, sites 7535 and 7553. Specifically for CIN3+ discrimination, a balanced sensitivity vs. specificity (68.1%, 66.2% respectively) with positive predictive value (PPV) and negative predictive value (NPV) (66.2%,68.2% respectively) was achieved for *UTR* 7535 methylation of 6.1% cutoff with overall accuracy 67.1%, while for *UTR* 7553 a sensitivity 60.9%, specificity 69.0%, PPV 65.6%, NPV 64.5% and overall accuracy 65.0% at threshold 10.1% was observed. For the remaining methylation sites we also calculated the thresholds that maximized overall accuracy or produced a balanced sensitivity vs. specificity. However, results produced were not performing well (data not shown).

## Discussion

Many epigenetic alterations are observed including DNA hypomethylation, hypermethylation of tumour suppressor genes and histone modifications during all the stages of cervical cancer 18. One of the epigenetic mechanisms that are increasingly studied is HPV genes' methylation. At present, a consistent correlation of increased methylation of capsid viral genes with histology severity is referred by the researchers 7, 9, 11, 19, 20. On the other hand, studies on methylation status of the *UTR*, *E6* and *E7* regions reveal heterogeneous and rather inconclusive results 21-25.

DNA methylation has been recognized as a frequent event in cervical cancer and as such, is referred as of valuable tool in the early detection of precancerous disease. In the present study we attempted to elucidate the methylation profile of HPV 16 genome for each of 4 CpG sites of the 5' *UTR*, 2 of the enhancer, 6 of the promoter, 3 of the *E6* and 3 of the *E7* gene in clinical specimens of different severities in a Greek women population. The studied sites were located in the 5' *UTR* at nt 7270, 7461, 7455 and 7428, in the enhancer at 7535, 7553, in the promoter at 7862, 31, 37, 43, 52, 58, in *E6* at 494, 502, 506 and in *E7* at 765, 780, 790.

The HPV16 *UTR* plays an important role in regulation of viral gene expression. HPV 16 *E6* and *E7* oncogenes are transcribed from the P97 promoter which is located at 3' *UTR* and is regulated by products of the viral E2 gene through a feedback mechanism. The transcription of the viral oncogenes depends also on the enhancer's activity, which is located between the positions 7454 and 7854 and acts as a cis-acting element that drives the transcription of the early HPV genes 26. Several host transcription factors, such as like AP-1, NF1, SP1, TFIID, TF1, Oct-1 are bound to specific sites of this viral gene triggering the over-production of *E6* and *E7* oncoproteins gradually leading to neoplastic progression 27, 28. There are studies supporting that this region is highly or moderately methylated 29-32 and the methylation is associated with the severity of cervical neoplasia. It is assumed that if E2 viral protein does not manage to bind at specific sites due to inhibition by methylation of cytosines within its binding site, the repression of oncogenes' transcription will be diminished. On the other hand, several studies claim that this region has an overall low percent methylation and there is no correlation of methylation with different severities of cervical carcinogenesis 10, 21, 24, 33, 34. According to the results of the present study, the mean methylation of HPV 16 *UTR* showed constantly low methylation percentages between the different histological groups. The only sites with remarkable results were 7535 and 7553 mapped at the enhancer, were a correlation with CIN 3+ can be proposed. These sites are part of the binding positions of NF-1 22, 35, 36. One could assume that such an increase of methylation could eliminate the NF-1 binding activity, affecting the enhancer's cis-acting efficiency for the transcription of the early HPV genes. But according to our results, although these specific enhancer sites are methylated along with the progression of the disease, we can probably suggest that the massive production of *E6*, *E7* oncoproteins is not affected by the addition of methyl groups at these sites and they may be selectively used to discriminate CIN3+ cases.

In the present study we also investigated the methylation of CpG sites that are located at *E6* and *E7* viral genes and specifically in those regions that are considered to be immunostimulatory motifs 25. According to Hacker et al 37, a sequence motif that contains CpGs has the capacity to stimulate certain immune cells. As far as HPV 16 is concerned it has been shown that TLR9 is capable of recognizing a CpG motif between nt 496 and 514 of *E6* gene 38 and methylation of the sites located into this genomic part could possibly lead to an escape from immune surveillance, having thus a significant biological impact in cervical carcinogenesis. One of the objectives of Sen et al. 25, was the analysis of the influence of methylation within two immunostimulatory CpG motifs within HPV16 *E6* and *E7* genes to cervical carcinogenesis. Presence of elevated methylation was shown at cervical cancer samples, with higher proportions at samples that had an integrated HPV 16 infection. In the present study, although we do not have the information concerning the integration of viral genome to the host genome, the percentages of methylation at CpG sites of *E6* and *E7* genes were elevated at the cervical cancer histology group when compared to precancer samples a finding that is in accordance to published results. As of statistical analysis revealed, these specific sites originated from *E6* and *E7* gene, cannot be proposed as biomarkers that could distinguish precancerous HPV 16 infections that have a true oncogenic potential from those that will dissolve without leading to disease.

In conclusion, the knowledge of viral genome's alterations during the viral life cycle is adding valuable information on understanding the biology of cervical cancer and to the exploring of new biomarkers. Frequently, different methodological approaches to the methylation study of viral genes may lead to inconsistent results, so the scientific community should feed the literature with findings of this area of HPV research. According to our results, methylation of HPV 16 *UTR* is not highly associated with severity of cervical neoplasm. However, two specific sites mapped at the enhancer region of *UTR*, could probably act as biomarkers and molecular determinants that could distinguish the rare HPV 16 infections that have a true oncogenic malignant potential from those common infections from HPV 16 that resolve spontaneously without leading to disease. Although a modest number of samples were studied, the reproducibility of these results should be assessed in large validation sets. Future studies should also analyze serial samples from larger cohorts to further assess the value of methylation as a predictive and diagnostic molecular determinant.

## Figures and Tables

**Figure 2:**
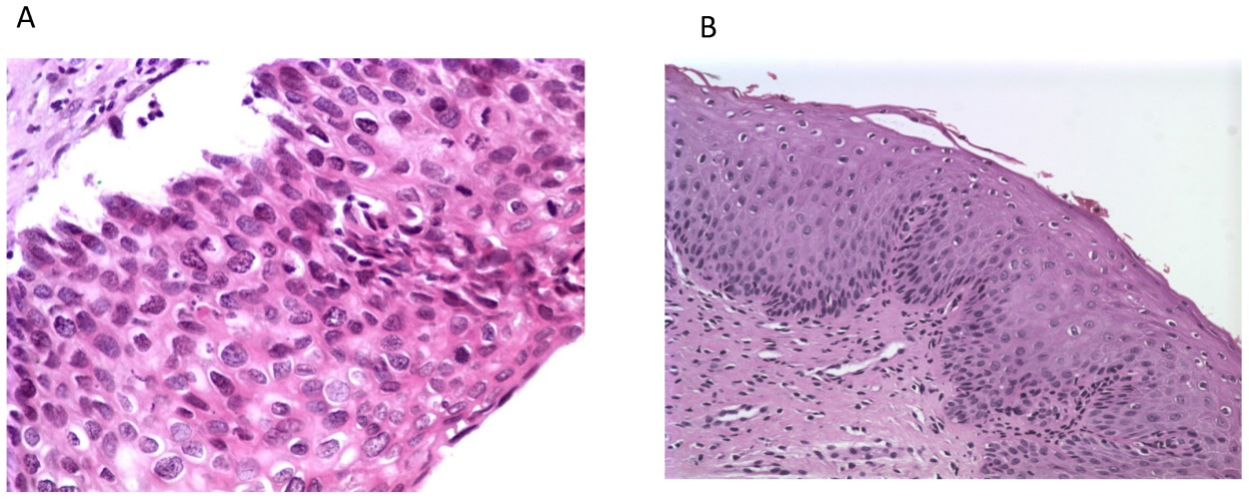
Histological photos from a CIN3 case (A, magnification 40x) with increased *UTR* 7535, 7553 sites HPV16 methylation and from a CIN1 case (B, magnification 10x) with absence of methylation at the same genomic viral sites.

**Table 1:** Primer sequences and CpGs sites that were studied

**UTR**	
Forward	GTGTGTTTGTATGTATGGTATA
Reverse	ATACAATAAATAACCACAACACAATTAA-BTN
Sequencing	GTGTGTTTGTATGTATGGTATA
Sequenced CpGs:	7270
Amplicon size:	133bp
Forward	TGTAGGTTAGGAAAATAGGGAT
Reverse	ACTATATTTACTACATCCTATTTTTATT-BTN
Sequencing	TTGTGTTAAAAAGTATGTAAT
	TAGGAAAATAGGGATTTGG
Sequenced CpGs:	7428, 7455, 7461
	7535, 7553
Amplicon size:	205bp
Forward	GTAAAATTGTATATGGGTGTGTGTAAA
Reverse	AATCCTAAAACATTACAATTCTCTTT-BTN
Sequencing	AATAATTTATGTATAAAATTAAGGG
	GTATATGGGTGTGTGTAAAT
Sequenced CpGs:	31, 37, 43, 52, 58
	7862
Amplicon size:	189 bp
**E6**	
Forward	ATGGGAATTTATATGTTGTATGTGAT
Reverse	CTCCTCCTCTAAACTATCATTTAATTACTC-BTN
Sequencing	AGATTTTATAATATAAGGGGT
Sequenced CpGs:	494, 502, 506
Amplicon size:	397 bp
**E7**	
Forward	ATGAAATAGATGGTTTAGTTGGATAAG-BTN
Reverse	AACACACAATTCCTAATATACCCATTAACA
Sequencing	CCATTAACAAATCTTCCAAAAT
Sequenced CpGs:	765, 780, 790
Amplicon size:	168 bp

**Table 2:** Distribution of single (HPV16 only) and multiple HPV infections of the studied population, in relation to the histological groups and the age using as cut-off the age of 30 years.

	Multiple Infections				Single Infection			
Histology/age group	<=30	>30	Subtotal		<=30	>30	Subtotal	Total
Negative		5	5 (71.4%)		1	1	2 (28.5%)	7 (4.8%)
CIN-1	7	7	14 (60.8%)		3	6	9 (39.1%)	23 (15.8%)
CIN-2	10	14	24 (53.3%)		8	13	21 (46.6%)	45 (31.0%)
CIN-3	13	19	32 (52.4%)		7	22	29 (47.5%)	61 (42.0%)
SCC		1	1 (16.6%)			5	5 (83.3%)	6 (4.1%)
Adenocarcinoma						3	3 (100%)	3 (2.0%)
**Total**	30 (39.4%)	46 (60.5%)	76 (52.4%)		19 (27.5%)	50 (72.4%)	69 (47.5%)	145

**Table 3:** Mean methylation and standard deviation according to the histological status and methylation site, grouped according to HPV region.

Region	Site	Normal (N=7)	CIN1 (N=23)	CIN2 (N=45)	CIN3 (N=61)	SCC (N=6)	AdenoCa (N=3)
**5'*UTR***	7270	11.1±11.8	12.4±14.9	14.0±10.0	14.7±10.2	20.0±20.6	28.0±17.0
	7461	3.7±2.3	2.6±1.3	2.7±1.1	4.3±2.5	12.0±10.1	10.0±9.5
	7455	4.2±0.9	3.1±1.4	3.4±1.1	4.1±4.2	14.2±16.0	11.3±9.0
	7428	3.2±1.2	2.9±1.6	3.1±1.6	3.4±1.5	11.0±8.6	9.0±6.5
	Mean	5.6±3.4	5.3±4.0	5.8±2.5	6.6±3.3	13.3±13.1	14.5±8.3
**Enhancer**	7535	9.4±5.5	8.1±6.1	6.3±3.2	11.1±8.4	27.6±14.1	21.3±10.0
	7553	11.1±9.5	12.0±9.1	10.1±5.5	14.6±11.2	39.2±19.2	21.3±5.0
	Mean	10.2±7.2	10.10±7.4	8.2±4.1	12.9±9.0	33.4±17.3	21.3±7.5
**Promoter**	7862	2.8±0.9	2.4±1.2	1.7±0.6	1.7±0.8	3.1±1.7	3.6±2.0
	31	1.4±0.5	2.2±1.0	2.3±1.7	1.8±1.3	7.5±9.9	9.0±11.3
	37	2.8±1.5	2.3±1.2	3.0±1.6	2.6±1.9	6.8±9.2	9.0±10.4
	43	2.0±1.0	2.3±1.1	2.2±3.6	1.4±1.2	2.6±3.2	9.3±12.7
	52	2.4±1.5	1.8±0.9	2.2±1.5	1.9±1.4	7.0±9.2	9.6±13.2
	58	2.8±2.4	2.3±1.2	2.4±1.7	2.2±1.5	7.0±10.3	9.6±11.5
	Mean	2.4±1.0	2.2±0.7	2.3±1.3	1.9±1.0	5.6±6.8	8.3±10.2
***E6***	494	8.4±7.5	8.3±6.9	8.8±7.8	10.2±8.3	25.3±16.1	12.6±12.4
	502	12.0±11.9	13.7±12.0	14.3±13.5	16.2±12.3	40.6±27.8	18.0±14.0
	506	8.0±6.9	8.1±6.7	8.2±7.9	9.2±8.2	25.3±17.1	11.0±11.3
	Mean	9.4±8.7	10.0±8.4	10.4±9.6	11.9±9.5	30.4±20.1	13.8±12.5
***E7***	765	10.0±4.8	8.0±3.9	10.3±6.9	10.2±6.8	29.6±17.2	4.5±2.1
	780	5.0±2.4	5.3±3.1	5.3±2.9	5.5±4.1	23.6±11.3	4.5±2.1
	790	3.6±1.8	5.6±3.9	5.0±3.0	6.2±5.3	24.0±13.3	3.0±0.0
	Mean	6.2±2.7	6.3±3.4	6.9±3.9	7.3±5.0	25.7±13.7	4.0±0.0

**Table 4:** Performance characteristics for discriminating CIN3+ cases using two cut-offs, one that maximizes overall accuracy (Optimal threshold) and one that balances sensitivity vs. specificity (Balanced threshold).

	Site 7535		Site 7553	
	Optimal threshold	Balanced threshold	Optimal threshold	Balanced threshold
Sensitivity	68.1	68.1	75.3	60.8
Specificity	66.2	66.2	54.9	69.0
PPV	66.2	66.2	61.9	65.6
NPV	68.1	68.1	69.6	64.4
FPR	33.8	33.8	45.0	30.9
FNR	31.8	31.8	24.6	39.1
OA	67.1	67.1	65.0	65.0
OR	4.1	4.1	3.7	3.4

PPV: Positive Predictive Value, NPV: Negative Predictive Value, FPR: False Positive Rate, FNR: False Negative Rate, OA: Overall Accuracy, OR: Odds Ratio. Note that Optimal threshold and balanced threshold was the same for site 7535 while for site 7553 the optimal threshold resulted in high sensitivity at the cost of specificity.

## References

[1] zur Hausen H (2002). Papillomaviruses and cancer: from basic studies to clinical application. Nature reviews Cancer.

[2] Munoz N, Bosch FX, de Sanjose S, Herrero R, Castellsague X, Shah KV (2003). Epidemiologic classification of human papillomavirus types associated with cervical cancer. N Engl J Med.

[3] Leal SM Jr, Gulley ML (2017). Current and Emerging Molecular Tests for Human Papillomavirus-Related Neoplasia in the Genomic Era. J Mol Diagn.

[4] Minarovits J, Demcsak A, Banati F, Niller HH (2016). Epigenetic Dysregulation in Virus-Associated Neoplasms. Adv Exp Med Biol.

[5] Kuss-Duerkop SK, Westrich JA, Pyeon D (2018). DNA Tumor Virus Regulation of Host DNA Methylation and Its Implications for Immune Evasion and Oncogenesis.

[6] Nedjai B, Reuter C, Ahmad A, Banwait R, Warman R, Carton J (2018). Molecular progression to cervical precancer, epigenetic switch or sequential model?. Int J Cancer.

[7] Kottaridi C, Kyrgiou M, Pouliakis A, Magkana M, Aga E, Spathis A (2017). Quantitative Measurement of L1 Human Papillomavirus Type 16 Methylation for the Prediction of Preinvasive and Invasive Cervical Disease. J Infect Dis.

[8] Torres-Rojas FI, Alarcon-Romero LDC, Leyva-Vazquez MA, Ortiz-Ortiz J, Mendoza-Catalan MA, Hernandez-Sotelo D (2018). Methylation of the L1 gene and integration of human papillomavirus 16 and 18 in cervical carcinoma and premalignant lesions. Oncol Lett.

[9] Chaiwongkot A, Niruthisard S, Kitkumthorn N, Bhattarakosol P (2017). Quantitative methylation analysis of human papillomavirus 16L1 gene reveals potential biomarker for cervical cancer progression. Diagn Microbiol Infect Dis.

[10] Clarke MA, Wentzensen N, Mirabello L, Ghosh A, Wacholder S, Harari A (2012). Human papillomavirus DNA methylation as a potential biomarker for cervical cancer. Cancer Epidemiol Biomarkers Prev.

[11] Mirabello L, Schiffman M, Ghosh A, Rodriguez AC, Vasiljevic N, Wentzensen N (2013). Elevated methylation of HPV16 DNA is associated with the development of high grade cervical intraepithelial neoplasia. Int J Cancer.

[12] Badal V, Chuang LS, Tan EH, Badal S, Villa LL, Wheeler CM (2003). CpG methylation of human papillomavirus type 16 DNA in cervical cancer cell lines and in clinical specimens: genomic hypomethylation correlates with carcinogenic progression. J Virol.

[13] DiMaggio C (2013). SAS for epidemiologists: applications and methods.

[14] Harris M, Taylor J (2003). Medical statistics made easy.

[15] Varlatzidou A, Pouliakis A, Stamataki M, Meristoudis C, Margari N, Peros G (2011). Cascaded learning vector quantizer neural networks for the discrimination of thyroid lesions. Anal Quant Cytol Histol.

[16] Pouliakis A, Margari C, Margari N, Chrelias C, Zygouris D, Meristoudis C (2014). Using classification and regression trees, liquid-based cytology and nuclear morphometry for the discrimination of endometrial lesions. Diagnostic cytopathology.

[17] Zygouris D, Pouliakis A, Margari N, Chrelias C, Terzakis E, Koureas N (2014). Classification of endometrial lesions by nuclear morphometry features extracted from liquid-based cytology samples: a system based on logistic regression model. Anal Quant Cytopathol Histpathol.

[18] Lu Q, Ma D, Zhao S (2012). DNA methylation changes in cervical cancers. Methods Mol Biol.

[19] Yang-Chun F, Yuan Z, Cheng-Ming L, Yan-Chun H, Xiu-Min M (2017). Increased HPV L1 gene methylation and multiple infection status lead to the difference of cervical epithelial cell lesion in different ethnic women of Xinjiang, China. Medicine (Baltimore).

[20] Sun C, Reimers LL, Burk RD (2011). Methylation of HPV16 genome CpG sites is associated with cervix precancer and cancer. Gynecol Oncol.

[21] Fernandez AF, Rosales C, Lopez-Nieva P, Grana O, Ballestar E, Ropero S (2009). The dynamic DNA methylomes of double-stranded DNA viruses associated with human cancer. Genome Res.

[22] Ding DC, Chiang MH, Lai HC, Hsiung CA, Hsieh CY, Chu TY (2009). Methylation of the long control region of HPV16 is related to the severity of cervical neoplasia. Eur J Obstet Gynecol Reprod Biol.

[23] Milutin Gasperov N, Sabol I, Planinic P, Grubisic G, Fistonic I, Corusic A (2015). Methylated Host Cell Gene Promoters and Human Papillomavirus Type 16 and 18 Predicting Cervical Lesions and Cancer. PLoS One.

[24] Kalantari M, Calleja-Macias IE, Tewari D, Hagmar B, Lie K, Barrera-Saldana HA (2004). Conserved methylation patterns of human papillomavirus type 16 DNA in asymptomatic infection and cervical neoplasia. J Virol.

[25] Sen S, Mandal P, Bhattacharya A, Kundu S, Roy Chowdhury R, Mondal NR (2017). Impact of viral and host DNA methylations on HPV16-related cervical cancer pathogenesis. Tumour Biol.

[26] Cripe TP, Haugen TH, Turk JP, Tabatabai F, Schmid PG 3rd, Durst M (1987). Transcriptional regulation of the human papillomavirus-16 E6-E7 promoter by a keratinocyte-dependent enhancer, and by viral E2 trans-activator and repressor gene products: implications for cervical carcinogenesis. EMBO J.

[27] Desaintes C, Demeret C (1996). Control of papillomavirus DNA replication and transcription. Semin Cancer Biol.

[28] Thierry F, Howley PM (1991). Functional analysis of E2-mediated repression of the HPV18 P105 promoter. New Biol.

[29] Cheung JL, Cheung TH, Yu MY, Chan PK (2013). Virological characteristics of cervical cancers carrying pure episomal form of HPV16 genome. Gynecol Oncol.

[30] Bhattacharjee B, Sengupta S (2006). CpG methylation of HPV 16 LCR at E2 binding site proximal to P97 is associated with cervical cancer in presence of intact E2. Virology.

[31] Thain A, Jenkins O, Clarke AR, Gaston K (1996). CpG methylation directly inhibits binding of the human papillomavirus type 16 E2 protein to specific DNA sequences. J Virol.

[32] Kim K, Garner-Hamrick PA, Fisher C, Lee D, Lambert PF (2003). Methylation patterns of papillomavirus DNA, its influence on E2 function, and implications in viral infection. J Virol.

[33] Brandsma JL, Sun Y, Lizardi PM, Tuck DP, Zelterman D, Haines GK 3rd (2009). Distinct human papillomavirus type 16 methylomes in cervical cells at different stages of premalignancy. Virology.

[34] Kalantari M, Garcia-Carranca A, Morales-Vazquez CD, Zuna R, Montiel DP, Calleja-Macias IE (2009). Laser capture microdissection of cervical human papillomavirus infections: copy number of the virus in cancerous and normal tissue and heterogeneous DNA methylation. Virology.

[35] Apt D, Chong T, Liu Y, Bernard HU (1993). Nuclear factor I and epithelial cell-specific transcription of human papillomavirus type 16. J Virol.

[36] Chong T, Apt D, Gloss B, Isa M, Bernard HU (1991). The enhancer of human papillomavirus type 16: binding sites for the ubiquitous transcription factors oct-1, NFA, TEF-2, NF1, and AP-1 participate in epithelial cell-specific transcription. J Virol.

[37] Hacker G, Redecke V, Hacker H (2002). Activation of the immune system by bacterial CpG-DNA. Immunology.

[38] Hasan UA, Bates E, Takeshita F, Biliato A, Accardi R, Bouvard V (2007). TLR9 expression and function is abolished by the cervical cancer-associated human papillomavirus type 16. J Immunol.

